# Trends in ischemic heart disease mortality among older adults with co-existing colorectal cancer in the US (1999–2023): a 25-Year retrospective study

**DOI:** 10.1186/s43044-025-00695-3

**Published:** 2025-10-14

**Authors:** Muhammad Shaheer Bin Faheem, Muhammad Mujtaba Shahid Baig, Rimsha Zafar, Syed Tawassul Hassan, Arfa Ahmed Assad, Talha Ali, Nafila Zeeshan, Faheem Feroze

**Affiliations:** 1 Karachi Institute of Medical Sciences, KIMS, Karachi, Pakistan; 2 CMH Multan Institute of Medical Sciences, CIMS, Multan, Pakistan; 3 Nishtar Medical College, Multan, Pakistan; 4 Karachi Medical and Dental College, Karachi, Pakistan; 5 Allama Iqbal Medical College, Lahore, Pakistan; 6 Shaheed Mohtarma Benazir Bhutto Medical College, Karachi, Pakistan; 7 Combined Military Hospital, CMH Rawalpindi, Rawalpindi, Pakistan

**Keywords:** Ischemic heart disease, Colorectal cancer, CDC Wonder, Mortality, Gender and racial disparities

## Abstract

**Introduction:**

Ischemic heart disease (IHD) continues to be the primary cause of death among older adults in the United States (U.S.), while colorectal cancer (CRC), being the 3rd most common cancer, contributes to increased mortality when co-occurs with IHD. This study examines national trends in IHD mortality from 1999 to 2023 among older adults with co-existing CRC.

**Methods:**

The study retrospectively analyzed death certificates from the CDC’s Wide-ranging ONline Data for Epidemiologic Research (WONDER) database, including older adults (aged ≥ 65 years) who listed IHD as the underlying cause of death while CRC was a contributing cause. Crude and age-adjusted mortality rates (AAMRs) per 100,000 individuals, as well as the annual percent change (APC) in AAMRs with a 95% confidence interval, were obtained.

**Results:**

A total of 43,417 deaths were attributed to IHD among patients with coexisting CRC. AAMR declined significantly throughout the study, being more prominent from 2003 to 2016 (APC − 8.65; *p* < *0.05*). Males (6.3) had doubled the AAMR that of females (3.1). Non-Hispanic (NH) African Americans represented the highest AAMR (4.5), followed by other races/ethnicities. Regionally, peak AAMR was observed in the northeast (5.7) and non-metropolitan areas (4.7).

**Conclusions:**

A substantial (75%) reduction in AAMR was observed from 1999 to 2023. However, disparities across different demographical and geographical highlight the need for targeted interventions to lower IHD burned among patients with co-existing CRC.

**Supplementary Information:**

The online version contains supplementary material available at 10.1186/s43044-025-00695-3.

## Introduction

In the U.S, IHD is the primary cause of death, particularly among older adults. According to the CDC wonder database, IHD accounted for over 371,506 deaths in 2022, representing a substantial burden on the healthcare system [1]. Similarly, CRC is the third most commonly diagnosed cancer and the third most common cause of cancer-related death in both men and women in the U.S. However, it ranks second in cancer-related deaths worldwide and is the leading cause of cancer-related death in men under the age of fifty [2]. According to World Health Organization, by 2040, the global burden of CRC is expected to rise to 3.2 million new cases annually and 1.6 million deaths annually [3]. These statistics are alarming, considering the growing burden they impose on society.

Although these conditions significantly contribute to the mortality burden independently, patients with IHD have a significantly increased risk of developing CRC [4], with recent studies showing that cardiovascular risk factors such as obesity, diabetes mellitus, hypertension, coronary artery disease, smoking and physical inactivity are associated with an increased risk of CRC [5–7]. These shared risk factors contribute to the development of both IHD and CRC and display common underlining mechanisms involving chronic inflammation, metabolic dysfunction and poor lifestyle patterns [7–9]. Conversely, cardiovascular diseases (CVD)s are the most common non-cancerous cause of death among older patients with CRC and long-term survivors [10, 11], highlighting a strong bidirectional relation between both the conditions beyond their common risk profiles. Furthermore, chemotherapies such as fluoropyrimidines used for CRC patients increase the risk of CVDs, particularly IHD and HF [5, 12], while pre-existing CVDs and associated risk factors lead to adverse CRC treatment outcomes and survival rates [12, 13].

Additionally, the combined burden of IHD and CRC is significantly prevalent among older adults (aged ≥ 65 years) due to the presence of comorbidities and age-related physiological decline, leading to high mortality rates and worse clinical outcomes [5, 6, 12]. This rising burden highlights the need to examine long-term IHD mortality trends among older adults with co-existing CRC, and therefore, we conducted a thorough analysis of the CDC WONDER database from 1999 to 2023 to determine IHD and CRC mortality trends across various geographical and demographical subgroups and inform future interventions related to cardiovascular and oncologic care.

## Methods

### Study setting and population

This study retrospectively analyzed death records of older adults (aged ≥ 65 years), listing IHD as the underlying cause of death while CRC as the contributing cause, covering all 50 states and the District of Columbia [14]. Datasets were gathered from the CDC WONDER Multiple Cause of Death database, which contains de-identified death certificate information for U.S. residents coded using the International Classification of Diseases, Tenth Revision (ICD-10). IHD and CRC mortalities were identified through the ICD 10 codes I20-I25 and C18-C21, respectively. The study utilized publicly available de-identified datasets and was therefore exempted from institutional review board (IRB) approval. Further, the study focused on older adults (≥ 65 years) because these conditions were uncommon in younger populations.

### Data extraction

The mortality data was categorized by population size, demographics including sex, race/ethnicity, place of death, and geographics comprising urbanization and census regions (Northeast, Midwest, South, and West). Race/ethnicity was classified based on U.S Census classifications and included NH White, NH African American, Hispanic/Latino, NH Asian/Pacific Islander, and NH American Indian/Alaska Native, while the degree of urbanization considered metropolitan and non-metropolitan areas following NCHS Urban–Rural Classification Scheme for Counties [15]. Further, the age group interval (65–85 + years) for older adults was uniformly applied across all stratifications according to the inclusion criteria, and mortality rates were age-adjusted to 2000 U.S. standard population to allow fair comparisons over time and between various demographical and geographical subgroups [16].

### Statistical analysis

We calculated the AAMRs and crude mortality rates (CMRs) per 100,000 older adult population. The number of relevant deaths was divided by the corresponding annual population to determine the CMRs. The direct method was used to standardize AAMRs using the U.S. 2000 normal population. Temporal trends were analyzed using Joinpoint Regression Analysis (V5.0, National Cancer Institute) [17], and APCs in AAMRs were calculated with their 95% CIs to detect statistically significant changes in trend segments over time across overall population and subgroups, including sex, race, and urbanization. [17]. Further, a maximum of four joinpoints was allowed while a data-driven weighted Bayesian information criterion (BIC) method was employed for model selection. A *p*-value < 0.05 was considered statistically significant.

## Results

### Proportional mortality rate across different variables from 1999 to 2023

Total 43,417 deaths were attributed to IHD among patients with co-existing CRC, representing 55.7% of males and 44.3% of females. Mortalities were highest in NH Whites (89.1%) followed by NH African American (8.5%), Hispanic or Latino (4%), NH Asian (2%). A large number of deaths occurred in in patient medical facilities (31.4%) with lesser frequencies in decedent's (26.8%), nursing home (25.9%), outpatient (9.6%) and other facilities (3.2%) while least (1.8%) recorded from hospice care. Regionally deaths were higher in metropolitan areas (80.5%) than in non-metropolitan areas (19.5%). South region accounted for the highest number of deaths (28.8%) as compared to Northeast (27.3%), Midwest (23.9%) and West (20%) regions respectively (Supplemental Table 1).

### Overall age-adjusted trends for ischemic heart disease-related mortality in older adults with colorectal cancer from 1999 to 2023

**Fig. 1:**
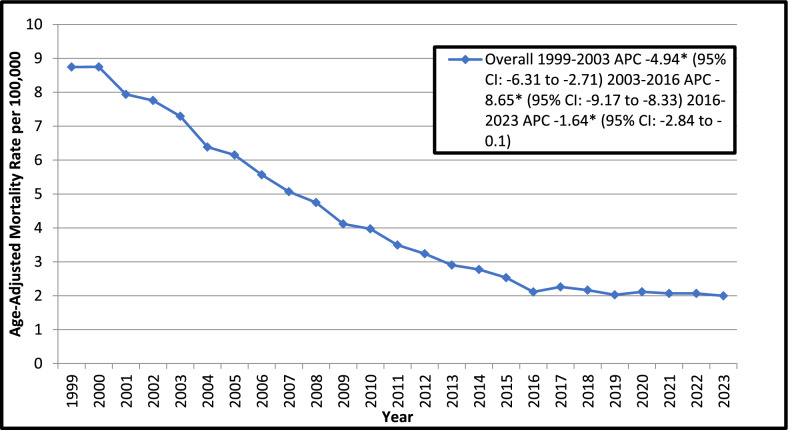
Overall Trends in Ischemic Heart Disease-related Age- Adjusted Mortality Rates in Older Adults with Colorectal Cancer in the United States, 1999 to 2023. APC = Annual Percentage Change, CI = Confidence Interval. *Indicates that the Annual Percentage Change (APC) is significantly different from zero at α = 0.05

The AAMR decreased to one fourth from 8.7 in 1999 to 2.0 in 2023. Significant declining trends were observed in AAMRs throughout the study period with a more prominent decline noted from 2003 to 2016 (APC: − 8.65; 95% CI: − 9.17 to − 8.33; *p* < *0.000001*) (Fig. 1) (Supplemental Tables 2, 3).

### Demographic trends

Notable variances were shown by AAMRs across different demographical subgroups.

#### Gender stratified

**Fig. 2:**
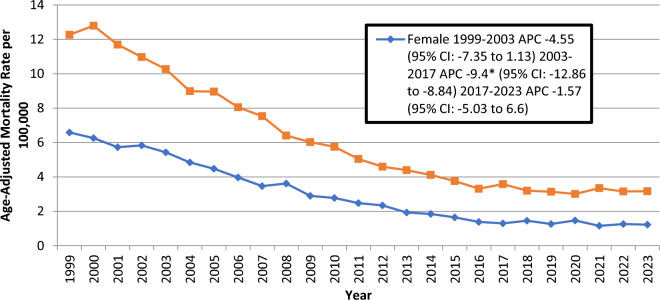
Trends in Ischemic Heart Disease-related Age- Adjusted Mortality Rates in Older Adults with Colorectal Cancer in the United States, 1999 to 2023, Stratified by Sex. APC = Annual Percentage Change, CI = Confidence Interval. *Indicates that the Annual Percentage Change (APC) is significantly different from zero at α = 0.05

The total AAMR reported by 2023 was 6.3 in males doubled that of females at 3.1. AAMR showed declining trends during our study in both genders, the trends being significant from 2001 to 2016 in males and from 2003 to 2017 in females with associated APCs of − 8.11 (95% CI: − 12.81 to − 4.94; *p* = *0.023195*) and − 9.4 (95% CI: − 12.86 to − 8.84; *p* = *0.000800*) respectively (Fig. 2) (Supplemental Tables 2, 3).

#### Race/ethnicity stratified

**Fig. 3:**
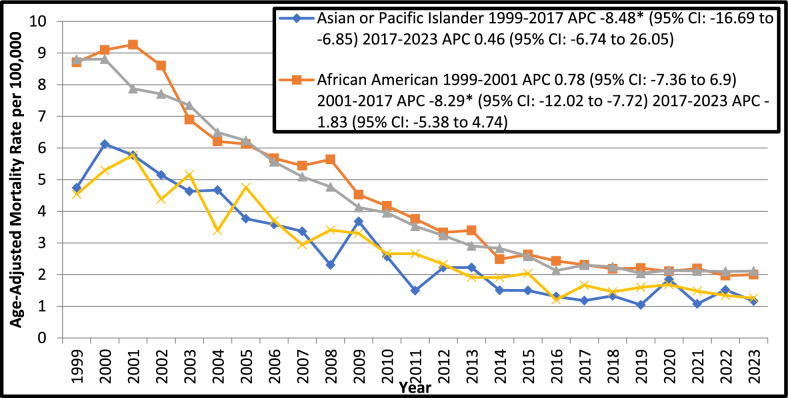
Trends in Ischemic Heart Disease-related Age- Adjusted Mortality Rates in Older Adults with Colorectal Cancer in the United States, 1999 to 2023, Stratified by Race. APC = Annual Percentage Change, CI = Confidence Interval. *Indicates that the Annual Percentage Change (APC) is significantly different from zero at α = 0.05

The recorded mortality rates were highest inNH African Americans (4.5) followed by NH Whites (4.4), Hispanics (2.9) and NH Asians or Pacific Islanders (2.8). From 1999 the AAMR declined significantly up to 2016 in NH Whites (APC *p* < *0.05*: 1999–2003: − 4.9; 95% CI: − 6.5 to − 1.9; 2003–2016: − 8.61; 95% CI: − 9.33 to − 8.24) and until 2017 in NH Asians or Pacific Islanders and NH African Americans with associated APCs of − 8.48 (95% CI: − 16.69 to − 6.85; *p* = *0.023595*) and − 8.29 (95% CI: 95% CI: − 12.02 to − 7.72; *p* = *0.013197*) respectively. However, In Hispanics the AAMR declined sharply throughout the study period (APC: − 6.34; 95% CI: − 7.19 to − 5.55; *p* < *0.000001*) (Fig. 3).

### Geographic trends

**Fig. 4:**
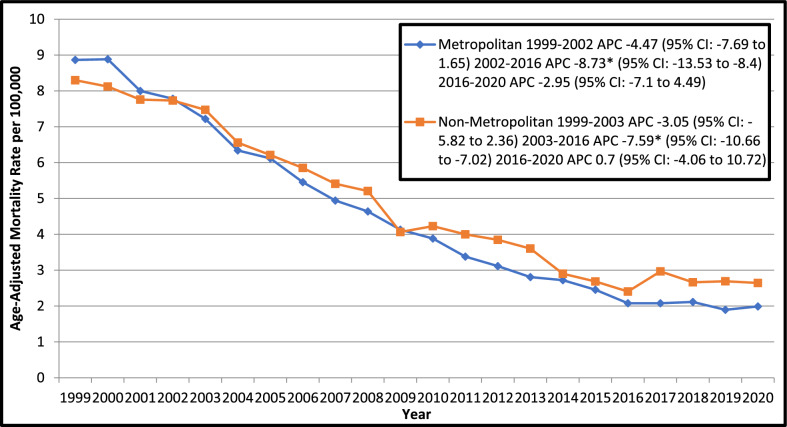
Trends in Ischemic Heart Disease-related Age- Adjusted Mortality Rates in Older Adults with Colorectal Cancer in the United States, 1999 to 2020, Stratified by Urban–Rural Classification. APC = Annual Percentage Change, CI = Confidence Interval. *Indicates that the Annual Percentage Change (APC) is significantly different from zero at α = 0.05

Significant disparities were found among geographical regions and states in top 90th percentile New York (7.8), Rhode Island (7.1), West Virginia (6.3), Iowa (5.8) and Ohio (5.7) had tripled the AAMRs of states Utah (1.6), Georgia (2.3), Nevada (2.4), Arizona (2.4) and Alabama (2.5) in lower 10th percentile. Further, AAMR was reported highest from the northeast region (5.7), followed by Midwest (4.5), West (4.2) and South (3.5) regions, respectively. Regionally higher AAMRs were showed by non-metropolitan areas (4.7) compared to metropolitan areas (4.3). However, AAMR decreased from 1999 to 2023 in both metropolitan and non-metropolitan areas, declining significantly from 2002 in metropolitan areas and from 2003 in non-metropolitan areas till 2016 with APCs of − 8.73 (95% CI: − 13.53 to − 8.4*; p* < *0.000001*) and − 7.59 (95% CI: − 10.66 to − 7.02; *p* < *0.000001*) respectively (Fig. 4).

## Discussion

Our retrospective analysis documents a sustained and substantial decline in age-adjusted mortality from IHD among older adults living with colorectal cancer (CRC) in the United States between 1999 and 2023. By 2023, the IHD-related AAMR had fallen to one-quarter of its 1999 level, mirroring national gains in cardiovascular prevention while revealing several demographic and geographic fault lines that warrant targeted attention.

Our analysis documents a marked reduction in overall mortality, which aligns with the well-established, nationwide trends in IHD-related mortality and general CVD mortality, which have decreased in the US in the last two decades [18, 19]. Therefore, the decline observed in our study may be a reflection of this overall improvement in cardiovascular outcomes rather than improvement in CRC-related treatment and advancements only. While addressing IHD-related mortality in CRC adults, three interlocking developments may have contributed to the steep, uninterrupted fall. Advancements in secondary prevention within oncology with cardio-oncology services, mandatory pre-chemotherapy cardiac risk assessments, and the routine use of statins and antiplatelets are now embedded in CRC care pathways and may have played an important role [20–22]. At the same time, the widespread adoption of guideline-directed medical therapies (GDMTs) for IHD, including high-intensity statins, dual antiplatelet therapy, and aggressive hypertension management, became common practice after the early 2000 s, further facilitated by Medicare Part D coverage for older adults [23, 24]. Moreover, contemporary CRC treatment strategies, such as oxaliplatin-sparing regimens for frailer patients and minimal-access surgery, have minimized peri-operative myocardial injury, a historically significant trigger for late IHD-related mortality [25, 26]. Collectively, these innovations may help mitigate the competing risk of IHD in CRC survivors.

Despite parallel downward trajectories, men finished the study period with a two-fold higher AAMR than women. As supported in the previous literature, many biological differences may contribute to these observed disparities, like males accumulating a heavier burden of atherogenic lipids and visceral adiposity in mid-life, which can increase their susceptibility to cardiovascular diseases. Furthermore, men are more likely to develop cardiovascular comorbidities at younger ages, which compounds their long-term risk [27, 28]. Lifestyle habits also play a crucial role in influencing health outcomes. Men, in particular, are more likely to engage in smoking and excessive alcohol consumption, both of which are linked to an increased risk of cardiovascular disease and cancer. While not directly assessed in this study, lifestyle differences like these not only exacerbate the progression of colorectal cancer but also accelerate the development of atherosclerosis and other cardiovascular complications, thereby significantly raising the risk of mortality [29–31]. Furthermore, men are less likely to receive GDMTs, such as statins and antiplatelet agents, due to concerns over potential interactions with cancer treatments and side effects like myopathy and gastrointestinal bleeding [32, 33]. Our findings suggest that cardio-oncology programs should incorporate sex-specific metrics to ensure that men experience the same absolute risk reductions as women, particularly through interventions tailored to better address gender-based disparities, as shown in this and prior literature [34].

The persistently elevated mortality in non-Hispanic African Americans, and, to a lesser extent, Whites, may reflect structural inequities that transcend oncology and highlight systemic barriers in healthcare. These disparities may be influenced by factors such as a higher prevalence of poorly controlled hypertension, limited access to preventive care, and a lower uptake of guideline-directed therapies. Additionally, delayed diagnosis and treatment of CRC in these populations exacerbate the risk of developing IHD. These issues may be compounded by socioeconomic factors such as limited healthcare access, lower health literacy, and racial bias in healthcare delivery [35–38]. Conversely, the sharper annual decline observed in Hispanics may be attributed to community-anchored prevention campaigns, and culturally tailored navigation services can have a meaningful impact on reducing mortality, as previously described in the literature. Programs that focus on improving education, outreach, and access to care within these communities appear to be effective, even when baseline services are limited [39]. This highlights the importance of addressing cultural and social determinants of health to bridge the gap in IHD and cancer-related mortality. Given that our study is descriptive, observational, these casualties aren’t explored here, and these interpretations should be viewed as hypothesis-generating. Further research is needed to confirm these associations and their underlying causes.

State-level outliers such as New York and Rhode Island exhibited AAMRs three times higher than Utah or Georgia, a spread too wide to be explained by case-mix alone. Two patterns emerge. First, the Northeast, despite its concentration of tertiary centers, showed the highest regional AAMR, reflecting studies that link densely populated urban corridors to higher environmental cardiotoxic exposures, limited space for green areas, and fragmented healthcare access [38, 40]. These factors, in combination, may contribute to heightened cardiovascular risks in urban populations. Second, non-metropolitan counties, though home to only one in five deaths, carried a heavier rate burden than metropolitan areas. Workforce shortages, limited access to rapid percutaneous coronary intervention, and the closure of rural cancer centers likely underpin this excess, compounded by travel barriers and longer times to treatment explored in other studies [41–43]. Tele-cardio-oncology pilots and mobile CRC screening units have shown early promise in closing these rural gaps, offering accessible care and detection, and merit scale-up to further reduce these disparities [44, 45].

These findings underscore the need for an explicitly intersectional cardio-oncology agenda. Interventions that couple evidence-based IHD therapy with CRC survivorship clinics should prioritize African American men, rural residents, and Northeastern states with stubbornly high rates. Embedding automated cardiovascular risk calculators into oncology electronic records, expanding pharmacist-led statin titration, and widening cardiac rehabilitation eligibility to include active cancer patients are pragmatic steps with immediate yield [28, 46, 47]. Finally, state cancer registries should be integrated with cardiovascular event databases to enable real-time surveillance and rapid-cycle quality improvement [48].

## Study limitations

When evaluating the results, it’s important to account for the limitations of this study. Firstly, the data used in this analysis is comprehensive and depends on the accuracy and completeness of death certificates, which may be subject to reporting errors or misclassification of either IHD or CRC, especially if it’s possible that CRC may be omitted from the death record if it’s not considered as the contributing cause leading to under ascertainment of deaths. Secondly, variations in coding practices and reporting standards may have changed over a 25-year study period and can cause discrepancies in trend analysis. Thirdly, small sample sizes of some subgroups may lead to low statistical precision estimates and large CIs. Furthermore, the database doesn’t provide any information related to the staging of cancer, the time since CRC diagnosis or the living CRC population at risk, which may influence the risk of IHD and the accuracy of observed mortality trends. Additionally, our study relied on secondary data, which limits our ability to control for potential confounders, such as the influence of lifestyle factors, comorbidities, and treatment regimens, which may also impact the observed mortality rates. Furthermore, while AAMRs were used to standardize results, this approach may not fully account for temporal or regional shifts in healthcare practices or diagnostic capabilities, which could influence trends in both IHD and CRC. Finally, the study’s cross-sectional nature limits causal inference; while correlations between IHD and CRC mortality are observed, the directionality of the relationship remains speculative, and further research using longitudinal designs is needed to confirm these findings.

## Conclusions

Based on the findings of this study, it’s evident that significant advancements in both cardiovascular and oncology care have contributed to a substantial reduction in age-adjusted mortality from IHD among older adults with coexistent CRC. However, notable disparities persist, particularly along demographic and geographic lines, highlighting the need for targeted interventions. Addressing sex-based differences, racial inequities, and regional healthcare access gaps is crucial in ensuring that all patients benefit equally from these advancements. Future efforts should focus on integrating cardio-oncology services with culturally tailored and geographically appropriate strategies to further reduce mortality and improve outcomes for CRC survivors.

## Supplementary Information

Additional file 1.

## Data Availability

Data is provided within the manuscript or supplementary information files.

## Abbreviations

IHD: Ischemic heart disease
US: United States
CRC: Colorectal cancer
CDC WONDER: Centers for Disease Control and Prevention Wide-Ranging Online Data for Epidemiologic Research
AAMR: Age-Adjusted Mortality Rates
APC: Annual Percent Change
IRB: Institutional Review Board
NCHS: National Center for Health Statistics
CMRs: Crude Mortality Rates
GDMTs: Guideline-directed medical therapies
