# Supplementary material for: Trends in ischemic heart disease mortality among older adults with co-existing colorectal cancer in the US (1999–2023): a 25-Year retrospective study
Source: Egypt Heart J. 2025 Oct 14;77:96. doi: 10.1186/s43044-025-00695-3 (PMC12521672; doi:10.1186/s43044-025-00695-3)
Supplement: Supplementary file 1 — Additional file 1. [file 43044_2025_695_MOESM1_ESM.docx]

**Supplemental Table 1:** Ischemic Heart Disease-related Age-Adjusted Mortality Rates per 100,000 in Older Adults with Colorectal Cancer, Overall and Stratified by Sex, in the United States, 1999 to 2023

| **Age-adjusted Mortality Rate (95% Confidence interval)** | | | |
| --- | --- | --- | --- |
| **Year** | **Females** | **Males** | **Overall** |
| 1999 | 6.587 (6.25 - 6.924) | 12.269 (11.641 - 12.897) | 8.743 (8.431 - 9.056) |
| 2000 | 6.255 (5.929 - 6.582) | 12.797 (12.162 - 13.432) | 8.747 (8.436 - 9.058) |
| 2001 | 5.728 (5.417 - 6.04) | 11.695 (11.092 - 12.298) | 7.939 (7.645 - 8.233) |
| 2002 | 5.832 (5.519 - 6.146) | 10.976 (10.398 - 11.555) | 7.757 (7.469 - 8.046) |
| 2003 | 5.433 (5.133 - 5.733) | 10.271 (9.719 - 10.823) | 7.293 (7.015 - 7.572) |
| 2004 | 4.844 (4.56 - 5.128) | 8.988 (8.476 - 9.5) | 6.386 (6.127 - 6.644) |
| 2005 | 4.476 (4.204 - 4.747) | 8.961 (8.456 - 9.467) | 6.152 (5.9 - 6.404) |
| 2006 | 3.967 (3.715 - 4.218) | 8.051 (7.581 - 8.522) | 5.567 (5.33 - 5.805) |
| 2007 | 3.468 (3.234 - 3.702) | 7.536 (7.088 - 7.985) | 5.07 (4.845 - 5.294) |
| 2008 | 3.615 (3.377 - 3.853) | 6.404 (5.999 - 6.809) | 4.748 (4.532 - 4.963) |
| 2009 | 2.898 (2.687 - 3.109) | 6.028 (5.638 - 6.418) | 4.116 (3.918 - 4.314) |
| 2010 | 2.775 (2.569 - 2.981) | 5.752 (5.375 - 6.128) | 3.971 (3.777 - 4.164) |
| 2011 | 2.477 (2.285 - 2.668) | 5.043 (4.695 - 5.39) | 3.494 (3.315 - 3.673) |
| 2012 | 2.342 (2.156 - 2.528) | 4.598 (4.273 - 4.922) | 3.24 (3.07 - 3.41) |
| 2013 | 1.929 (1.763 - 2.096) | 4.393 (4.08 - 4.705) | 2.905 (2.746 - 3.064) |
| 2014 | 1.846 (1.685 - 2.007) | 4.118 (3.82 - 4.417) | 2.774 (2.62 - 2.928) |
| 2015 | 1.647 (1.496 - 1.797) | 3.758 (3.478 - 4.038) | 2.533 (2.387 - 2.678) |
| 2016 | 1.381 (1.241 - 1.52) | 3.315 (3.055 - 3.576) | 2.114 (1.983 - 2.245) |
| 2017 | 1.297 (1.168 - 1.427) | 3.579 (3.311 - 3.846) | 2.262 (2.127 - 2.397) |
| 2018 | 1.452 (1.314 - 1.589) | 3.197 (2.952 - 3.443) | 2.165 (2.037 - 2.294) |
| 2019 | 1.266 (1.136 - 1.395) | 3.14 (2.9 - 3.38) | 2.028 (1.904 - 2.153) |
| 2020 | 1.467 (1.33 - 1.604) | 3.009 (2.777 - 3.241) | 2.115 (1.99 - 2.24) |
| 2021 | 1.153 (1.031 - 1.275) | 3.35 (3.097 - 3.602) | 2.07 (1.944 - 2.196) |
| 2022 | 1.261 (1.137 - 1.385) | 3.155 (2.918 - 3.391) | 2.068 (1.946 - 2.191) |
| 2023 | 1.222 (1.099 - 1.346) | 3.169 (2.933 - 3.404) | 1.997 (1.878 - 2.117) |
| Total | 3.06472 (2.8574 - 3.272) | 6.30208 (5.91656 - 6.68756) | 4.33016 (4.13488 - 4.52556) |

**Supplementary Figure 1:** Trends in Ischemic Heart Disease-related Age- Adjusted Mortality Rates in Older Adults with Colorectal Cancer in the United States, 1999 to 2020, Stratified by State


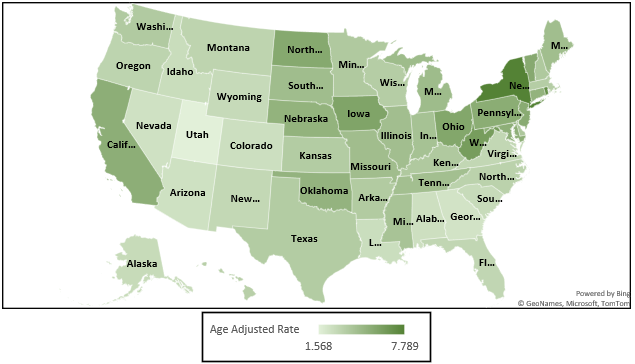


**Supplementary Figure 2:** Percent total deaths of ischemic heart disease-related by place of death among Older Adults with Colorectal Cancer in the United States, 1999 to 2023

**Supplemental Table 2:** Ischemic Heart Disease-related Age-Adjusted Mortality Rates per 100,000 in Older Adults with Colorectal Cancer, Stratified by Race, in the United States, 1999 to 2023

|  | **Age-Adjusted Mortality Rate (95% Confidence Interval)** | | | |
| --- | --- | --- | --- | --- |
| **Year** | **NH Asian or Pacific Islander** | **NH African American** | **NH White** | **Hispanic** |
| 1999 | 4.744 (3.201 - 6.773) | 8.706 (7.593 - 9.819) | 8.797 (8.467 - 9.127) | 4.535 (3.461 - 5.838) |
| 2000 | 6.125 (4.334 - 8.407) | 9.098 (7.962 - 10.235) | 8.808 (8.479 - 9.137) | 5.297 (4.167 - 6.64) |
| 2001 | 5.776 (4.163 - 7.807) | 9.265 (8.12 - 10.411) | 7.876 (7.568 - 8.185) | 5.775 (4.613 - 7.141) |
| 2002 | 5.149 (3.679 - 7.012) | 8.604 (7.509 - 9.7) | 7.706 (7.403 - 8.01) | 4.384 (3.418 - 5.539) |
| 2003 | 4.63 (3.293 - 6.33) | 6.9 (5.933 - 7.867) | 7.351 (7.057 - 7.646) | 5.159 (4.127 - 6.372) |
| 2004 | 4.669 (3.351 - 6.334) | 6.21 (5.295 - 7.125) | 6.495 (6.218 - 6.772) | 3.396 (2.61 - 4.345) |
| 2005 | 3.768 (2.639 - 5.217) | 6.127 (5.224 - 7.031) | 6.238 (5.969 - 6.507) | 4.755 (3.814 - 5.858) |
| 2006 | 3.587 (2.513 - 4.966) | 5.675 (4.813 - 6.536) | 5.565 (5.315 - 5.816) | 3.702 (2.891 - 4.67) |
| 2007 | 3.367 (2.358 - 4.661) | 5.443 (4.609 - 6.276) | 5.094 (4.856 - 5.333) | 2.941 (2.255 - 3.77) |
| 2008 | 2.306 (1.52 - 3.355) | 5.641 (4.803 - 6.479) | 4.772 (4.543 - 5.002) | 3.412 (2.688 - 4.271) |
| 2009 | 3.682 (2.686 - 4.927) | 4.525 (3.787 - 5.263) | 4.124 (3.913 - 4.336) | 3.303 (2.603 - 4.135) |
| 2010 | 2.573 (1.771 - 3.614) | 4.168 (3.466 - 4.87) | 3.955 (3.75 - 4.16) | 2.659 (2.043 - 3.401) |
| 2011 | 1.496 (0.926 - 2.287) | 3.759 (3.104 - 4.415) | 3.53 (3.339 - 3.722) | 2.661 (2.07 - 3.367) |
| 2012 | 2.223 (1.53 - 3.122) | 3.333 (2.736 - 3.93) | 3.237 (3.056 - 3.418) | 2.325 (1.798 - 2.958) |
| 2013 | 2.226 (1.559 - 3.082) | 3.396 (2.792 - 4) | 2.905 (2.735 - 3.075) | 1.913 (1.449 - 2.478) |
| 2014 | 1.504 (0.991 - 2.188) | 2.489 (2.016 - 3.039) | 2.831 (2.664 - 2.997) | 1.906 (1.465 - 2.439) |
| 2015 | 1.497 (1.003 - 2.151) | 2.636 (2.128 - 3.144) | 2.58 (2.422 - 2.739) | 2.039 (1.587 - 2.581) |
| 2016 | 1.306 (0.861 - 1.901) | 2.43 (1.948 - 2.913) | 2.127 (1.986 - 2.268) | 1.206 (0.876 - 1.618) |
| 2017 | 1.178 (0.769 - 1.726) | 2.31 (1.871 - 2.821) | 2.299 (2.153 - 2.445) | 1.666 (1.286 - 2.124) |
| 2018 | 1.331 (0.898 - 1.9) | 2.183 (1.748 - 2.618) | 2.25 (2.107 - 2.393) | 1.456 (1.106 - 1.882) |
| 2019 | 1.044 (0.682 - 1.529) | 2.207 (1.775 - 2.639) | 2.041 (1.907 - 2.174) | 1.597 (1.24 - 2.025) |
| 2020 | 1.855 (1.363 - 2.467) | 2.104 (1.692 - 2.515) | 2.139 (2.003 - 2.276) | 1.684 (1.312 - 2.127) |
| 2021 | 1.076 (0.715 - 1.556) | 2.195 (1.764 - 2.627) | 2.114 (1.975 - 2.254) | 1.477 (1.137 - 1.886) |
| 2022 | 1.518 (1.094 - 2.052) | 1.968 (1.573 - 2.362) | 2.101 (1.967 - 2.235) | 1.343 (1.027 - 1.725) |
| 2023 | 1.166 (0.797 - 1.646) | 1.998 (1.605 - 2.392) | 2.111 (1.976 - 2.246) | 1.257 (0.957 - 1.622) |
| Total | 2.79184 (1.94784 - 3.8804) | 4.5348 (3.83464 - 5.24108) | 4.36184 (4.15312 - 4.57092) | 2.87392 (2.24 - 3.63248) |

**Supplemental Table 3:** Ischemic Heart Disease-related Age-Adjusted Mortality Rates per 100,000 in Older Adults with Colorectal Cancer, Stratified by State, in the United States, 1999 to 2023

| **State** | **Age-adjusted Mortality Rate** |  |
| --- | --- | --- |
|  | 1999-2020 | |
| Utah | 1.568 | |
| Georgia | 2.284 | |
| Nevada | 2.397 | |
| Arizona | 2.442 | |
| Alabama | 2.504 | |
| Colorado | 2.526 | |
| Louisiana | 2.604 | |
| Idaho | 2.661 | |
| South Carolina | 2.736 | |
| Alaska | 2.765 | |
| Wyoming | 2.787 | |
| New Mexico | 2.933 | |
| Virginia | 2.99 | |
| Florida | 3.019 | |
| Montana | 3.149 | |
| Oregon | 3.179 | |
| North Carolina | 3.285 | |
| Hawaii | 3.412 | |
| Massachusetts | 3.528 | |
| Wisconsin | 3.534 | |
| Texas | 3.605 | |
| Kansas | 3.61 | |
| Arkansas | 3.686 | |
| Washington | 3.696 | |
| Kentucky | 3.774 | |
| Minnesota | 3.778 | |
| New Hampshire | 3.796 | |
| Delaware | 3.896 | |
| Mississippi | 4.1 | |
| Indiana | 4.178 | |
| Tennessee | 4.332 | |
| Illinois | 4.376 | |
| Maine | 4.41 | |
| Missouri | 4.437 | |
| South Dakota | 4.449 | |
| Michigan | 4.529 | |
| Oklahoma | 4.994 | |
| Nebraska | 5.039 | |
| Connecticut | 5.042 | |
| Maryland | 5.26 | |
| California | 5.281 | |
| Pennsylvania | 5.383 | |
| New Jersey | 5.478 | |
| District of Columbia | 5.491 | |
| Vermont | 5.555 | |
| North Dakota | 5.6 | |
| Ohio | 5.723 | |
| Iowa | 5.847 | |
| West Virginia | 6.261 | |
| Rhode Island | 7.068 | |
| New York | 7.789 | |

**Supplemental Table 4:** Ischemic Heart Disease-related Age-Adjusted Mortality Rates per 100,000 in Older Adults with Colorectal Cancer, Stratified by Urban-Rural Classification, in the United States, 1999 to 2020

|  | **Age-Adjusted Mortality Rate (95% Confidence Interval)** | |
| --- | --- | --- |
| Year | **Metropolitan** | **Nonmetropolitan** |
| 1999 | 8.864 (8.512 - 9.215) | 8.299 (7.609 - 8.988) |
| 2000 | 8.883 (8.534 - 9.232) | 8.121 (7.441 - 8.8) |
| 2001 | 7.999 (7.671 - 8.328) | 7.758 (7.095 - 8.42) |
| 2002 | 7.781 (7.459 - 8.102) | 7.732 (7.072 - 8.392) |
| 2003 | 7.221 (6.914 - 7.528) | 7.471 (6.825 - 8.118) |
| 2004 | 6.339 (6.054 - 6.625) | 6.559 (5.954 - 7.165) |
| 2005 | 6.12 (5.842 - 6.398) | 6.212 (5.628 - 6.795) |
| 2006 | 5.455 (5.195 - 5.715) | 5.853 (5.289 - 6.417) |
| 2007 | 4.944 (4.699 - 5.189) | 5.41 (4.873 - 5.948) |
| 2008 | 4.64 (4.405 - 4.875) | 5.208 (4.681 - 5.736) |
| 2009 | 4.129 (3.91 - 4.348) | 4.065 (3.601 - 4.528) |
| 2010 | 3.883 (3.672 - 4.094) | 4.229 (3.76 - 4.699) |
| 2011 | 3.381 (3.187 - 3.576) | 4 (3.548 - 4.451) |
| 2012 | 3.115 (2.931 - 3.3) | 3.848 (3.408 - 4.288) |
| 2013 | 2.808 (2.635 - 2.981) | 3.604 (3.182 - 4.026) |
| 2014 | 2.722 (2.554 - 2.889) | 2.903 (2.526 - 3.28) |
| 2015 | 2.456 (2.299 - 2.613) | 2.687 (2.331 - 3.044) |
| 2016 | 2.078 (1.934 - 2.221) | 2.406 (2.072 - 2.741) |
| 2017 | 2.078 (1.936 - 2.219) | 2.968 (2.598 - 3.339) |
| 2018 | 2.116 (1.975 - 2.257) | 2.662 (2.313 - 3.01) |
| 2019 | 1.896 (1.765 - 2.028) | 2.691 (2.346 - 3.037) |
| 2020 | 1.99 (1.857 - 2.123) | 2.644 (2.304 - 2.984) |
| Total | 4.271 (4.224 - 4.317) | 4.702 (4.598 - 4.807) |

**Supplemental Table 5:** Ischemic Heart Disease-related Age-Adjusted Mortality Rates per 100,000 in Older Adults with Colorectal Cancer, Stratified by Census Regions, in the United States, 1999 to 2023

|  | **Age-Adjusted Mortality Rate (95% Confidence Interval)** | | | |
| --- | --- | --- | --- | --- |
| **Year** | **Northeast** | **Midwest** | **South** | **West** |
| 1999 | 11.868 (11.084 - 12.652) | 9.151 (8.503 - 9.8) | 6.611 (6.149 - 7.074) | 8.521 (7.819 - 9.223) |
| 2000 | 11.295 (10.535 - 12.055) | 8.807 (8.175 - 9.44) | 7.11 (6.633 - 7.586) | 8.684 (7.982 - 9.386) |
| 2001 | 10.292 (9.571 - 11.013) | 8.4 (7.784 - 9.015) | 6.233 (5.788 - 6.677) | 7.888 (7.227 - 8.548) |
| 2002 | 10.496 (9.769 - 11.222) | 7.55 (6.969 - 8.131) | 6.44 (5.992 - 6.889) | 7.45 (6.813 - 8.087) |
| 2003 | 9.856 (9.158 - 10.554) | 7.454 (6.88 - 8.028) | 5.783 (5.361 - 6.206) | 6.863 (6.26 - 7.467) |
| 2004 | 8.574 (7.926 - 9.222) | 6.541 (6.004 - 7.077) | 5.089 (4.695 - 5.483) | 6.183 (5.616 - 6.749) |
| 2005 | 8.116 (7.489 - 8.744) | 6.287 (5.766 - 6.808) | 4.716 (4.342 - 5.09) | 6.286 (5.721 - 6.851) |
| 2006 | 6.985 (6.405 - 7.565) | 5.926 (5.421 - 6.431) | 4.29 (3.936 - 4.644) | 5.835 (5.296 - 6.373) |
| 2007 | 7.116 (6.536 - 7.696) | 5.065 (4.603 - 5.527) | 3.983 (3.648 - 4.317) | 4.586 (4.116 - 5.056) |
| 2008 | 6.584 (6.027 - 7.142) | 5.203 (4.738 - 5.668) | 3.555 (3.24 - 3.869) | 4.344 (3.893 - 4.795) |
| 2009 | 5.636 (5.124 - 6.147) | 3.902 (3.505 - 4.3) | 3.41 (3.105 - 3.714) | 3.987 (3.561 - 4.414) |
| 2010 | 5.24 (4.751 - 5.728) | 4.214 (3.798 - 4.629) | 3.117 (2.83 - 3.404) | 3.762 (3.348 - 4.176) |
| 2011 | 4.816 (4.35 - 5.282) | 3.636 (3.255 - 4.018) | 2.688 (2.425 - 2.951) | 3.363 (2.981 - 3.746) |
| 2012 | 4.445 (3.998 - 4.892) | 3.332 (2.969 - 3.695) | 2.698 (2.438 - 2.958) | 2.949 (2.597 - 3.302) |
| 2013 | 3.596 (3.203 - 3.989) | 3.273 (2.914 - 3.632) | 2.354 (2.113 - 2.594) | 2.894 (2.55 - 3.238) |
| 2014 | 3.973 (3.559 - 4.387) | 2.793 (2.462 - 3.124) | 2.182 (1.956 - 2.408) | 2.593 (2.274 - 2.913) |
| 2015 | 3.282 (2.908 - 3.656) | 2.748 (2.425 - 3.071) | 2.055 (1.838 - 2.272) | 2.359 (2.059 - 2.659) |
| 2016 | 2.784 (2.435 - 3.133) | 2.177 (1.896 - 2.458) | 1.739 (1.544 - 1.934) | 2.11 (1.83 - 2.389) |
| 2017 | 2.997 (2.644 - 3.351) | 2.333 (2.041 - 2.624) | 1.964 (1.758 - 2.169) | 1.948 (1.683 - 2.214) |
| 2018 | 2.689 (2.356 - 3.021) | 2.387 (2.096 - 2.677) | 1.878 (1.68 - 2.076) | 2.056 (1.789 - 2.322) |
| 2019 | 2.636 (2.311 - 2.961) | 2.172 (1.896 - 2.447) | 1.759 (1.57 - 1.948) | 1.813 (1.565 - 2.061) |
| 2020 | 2.73 (2.402 - 3.058) | 1.928 (1.668 - 2.187) | 1.873 (1.68 - 2.066) | 2.221 (1.948 - 2.494) |
| 2021 | 2.247 (1.944 - 2.549) | 2.082 (1.807 - 2.356) | 2.03 (1.826 - 2.235) | 1.95 (1.69 - 2.21) |
| 2022 | 2.493 (2.186 - 2.8) | 1.958 (1.701 - 2.215) | 1.91 (1.717 - 2.103) | 2.042 (1.787 - 2.298) |
| 2023 | 2.097 (1.815 - 2.379) | 2.043 (1.78 - 2.306) | 1.986 (1.792 - 2.179) | 1.985 (1.734 - 2.237) |
| Total | 5.71372 (5.21944 - 6.20792) | 4.45448 (4.04224 - 4.86656) | 3.49812 (3.20224 - 3.79384) | 4.18688 (3.76556 - 4.60832) |

**Supplemental Table** **6:** Annual Percentage Change of Ischemic Heart Disease-related Age-Adjusted Mortality Rates per 100,000 in Older Adults with Colorectal Cancer, in the United States, 1999 to 2023

| Year Interval | Annual Percentage Change (95% Confidence Interval) |
| --- | --- |
| Overall | |
| 1999-2003 | -4.94* (-6.31 - -2.71) |
| 2003-2016 | -8.65* (-9.17 - -8.33) |
| 2016-2023 | -1.64* (-2.84 - -0.1) |
| Females | |
| 1999-2003 | -4.55 (-7.35 - 1.13) |
| 2003-2017 | -9.4* (-12.86 - -8.84) |
| 2017-2023 | -1.57 (-5.03 - 6.6) |
| Males | |
| 1999-2001 | -1.97 (-7.7 - 3.89) |
| 2001-2016 | -8.11* (-12.81 - -4.94) |
| 2016-2023 | -1.21 (-3.68 - 3.73) |
| Metropolitan^a^ | |
| 1999-2002 | -4.47 (-7.69 - 1.65) |
| 2002-2016 | -8.73* (-13.53 - -8.4) |
| 2016-2020 | -2.95 (-7.1 - 4.49) |
| Nonmetropolitan ^a^ | |
| 1999-2003 | -3.05 (-5.82 - 2.36) |
| 2003-2016 | -7.59* (-10.66 - -7.02) |
| 2016-2020 | 0.7 (-4.06 - 10.72) |
| NH Asian or Pacific Islander | |
| 1999-2017 | -8.48* (-16.69 - -6.85) |
| 2017-2023 | 0.46 (-6.74 - 26.05) |
| NH African American | |
| 1999-2001 | 0.78 (-7.36 - 6.9) |
| 2001-2017 | -8.29* (-12.02 - -7.72) |
| 2017-2023 | -1.83 (-5.38 - 4.74) |
| NH White | |
| 1999-2003 | -4.9* (-6.5 - -1.9) |
| 2003-2016 | -8.61* (-9.33 - -8.24) |
| 2016-2023 | -1.3 (-2.74 - 0.7) |
| Hispanic | |
| 1999-2023 | -6.34* (-7.19 - -5.55) |

a. 1999-2020 data utilized only

*indicates that APC is significantly different from zero at the alpha = 0.05

NH indicates Non-Hispanic

**Supplemental Table 7:** Percentage total deaths for Ischemic Heart Disease-related Mortality in Older Adults with Colorectal Cancer, in the United States, 1999 to 2023

| Variable | Total Deaths (%) |
| --- | --- |
| Overall | 43417 (100) |
| Gender ^a^ |  |
| Male | 24193 (55.72) |
| Female | 19224 (44.28) |
| Census Region ^a^ |  |
| Northeast | 11839 (27.27) |
| Midwest | 10388 (23.93) |
| South | 12502 (28.80) |
| West | 8688 (20.01) |
| Race / Ethnicity ^a^ |  |
| NH Asian or Pacific Islander | 865 (1.99) |
| NH African American | 3714 (8.55) |
| NH White | 38687 (89.11) |
| Hispanic or Latino | 1728 (3.98) |
| Urbanization ^a,c^ |  |
| Metropolitan | 32327 (80.5) |
| Non metropolitan | 7833 (19.5) |
| Place of Death ^b^ |  |
| Inpatient Medical Facility | 13637 (31.41) |
| Outpatient Medical Facility | 4183 (9.63) |
| Decedent's Home | 11637 (26.8) |
| Hospice Facility | 799 (1.84) |
| Nursing Home/Long-term Care Facility | 11254 (25.92) |
| Other | 1367 (3.15) |

a Age Adjusted Mortality Rates (AAMRs) is utilized.

b Age Adjusted Mortality Rates (AAMRs) and AAPC is not applicable for Place of Death.

C: 1999-2020 data utilized only

*indicates that AAPC is significantly different from zero at the alpha = 0.05

NH indicates Non-Hispanic
